# Intralipid fails to rescue bupivacaine-induced cardiotoxicity in late-pregnant rats

**DOI:** 10.3389/fmed.2022.899036

**Published:** 2022-08-12

**Authors:** Caitlin Sherman, Natalie Koons, Michael Zargari, Catherine Cha, Jason Hirsch, Richard Hong, Mansoureh Eghbali, Soban Umar

**Affiliations:** ^1^Division of Molecular Medicine, Department of Anesthesiology and Perioperative Medicine, David Geffen School of Medicine at University of California, Los Angeles, Los Angeles, CA, United States; ^2^University of New England College of Osteopathic Medicine, Biddeford, ME, United States

**Keywords:** intralipid, bupivacaine, cardiotoxicity, pregnancy, resuscitation

## Abstract

**Background:**

Females routinely receive bupivacaine for obstetric and regional anesthesia. An accidental overdose of bupivacaine can result in cardiotoxicity and cardiac arrest. Intralipid (ILP) rescues bupivacaine-induced cardiotoxicity in male rats. However, bupivacaine cardiotoxicity and ILP rescue have not been studied in non-pregnant and late-pregnant female rats. Here, we tested the hypothesis that an appropriate dose of ILP would rescue non-pregnant and late-pregnant rats from bupivacaine-induced cardiotoxicity.

**Methods:**

Non-pregnant (*n* = 6) and late-pregnant (*n* = 7) female rats received intravenous bupivacaine (10-mg/kg bolus) to induce asystole. Resuscitation with 20% ILP (5-ml/kg actual body weight, single bolus, and 0.5-ml/kg/min maintenance) and chest compressions were continued for 10-min. Serial heart rate (HR), left ventricular ejection-fraction (LVEF%), and LV-fractional shortening (LVFS%) were recorded at baseline and 10-min after bupivacaine-induced cardiac arrest. Data are mean ± SD followed by 95% CI. *P*-values < 0.05 were considered statistically significant.

**Results:**

All rats developed cardiac arrest within a few seconds after bupivacaine. All non-pregnant rats were successfully rescued by ILP, with a HR of 280 ± 32 bpm at baseline vs. 212 ± 18 bpm at 10-min post ILP (*p* < 0.01), LVEF of 70 ± 6% vs. 68 ± 5% (*p* = ns), and LVFS of 41 ± 5% vs. 39 ± 4% (*p* = ns). Interestingly, 6 out of 7 late-pregnant rats did not recover with ILP. Baseline HR, LVEF and LVFS for late-pregnant rats were 330 ± 40 bpm, 66 ± 5% and 38 ± 4%, respectively. At 10-min post ILP, the HR, LVEF, and LVFS were 39 ± 102 bpm (*p* < 0.0001), 8 ± 22% (*p* < 0.0001), and 5 ± 12% (*p* < 0.001), respectively.

**Conclusions:**

ILP successfully rescued bupivacaine-induced cardiac arrest in non-pregnant rats, but failed to rescue late-pregnant rats.

## Introduction

Female patients routinely receive neuraxial anesthesia for labor and delivery. Bupivacaine is one of the most commonly used local anesthetics in obstetric and regional anesthesia. Some laboratory studies have suggested that pregnancy increases the cardiotoxicity of bupivacaine (1). An overdose of bupivacaine can result in cardiotoxicity and cardiac arrest (2–4). While the specific mechanism by which pregnancy causes increased susceptibility to local anesthetics is unknown, it is proposed that mechanical, hormonal, and biochemical changes may contribute to synergistic mechanisms to increase local anesthetic effectiveness. In previous studies, we and others have shown that intralipid (ILP) rescues the heart from bupivacaine-induced cardiotoxicity in male rats (2, 3, 5). However, surprisingly bupivacaine cardiotoxicity and ILP rescue have not been studied in female rats, defying the importance of sex as a biological variable in pre-clinical research.

During pregnancy, increased cardiac output likely alters uptake of local anesthetic and can affect the pharmacokinetics and pharmacodynamics of bupivacaine (4). The predisposition to acute central nervous system toxicity likely occurs due to autoregulation of cerebral blood flow. The amount of local anesthetic in the blood that travels to the brain is increased in patients with low cardiac output (4). It has been previously demonstrated that ILP protects the heart against ischemia/reperfusion (I/R) injury and bupivacaine-induced cardiotoxicity (3, 6, 7), and has also been shown to protect the heart in late-pregnancy against I/R injury (8). Interestingly and of specific interest to this study, ventricular hypertrophy abrogated ILP-induced cardioprotection against I/R injury by alteration of cardioprotective signaling (9).

Prior experimental studies on ILP rescue of bupivacaine cardiotoxicity have exclusively been performed on male rats without the inclusion of non-pregnant or pregnant females (2–5). As ILP has been shown to successfully rescue male rats with bupivacaine-induced cardiotoxicity, in the current study we tested the hypothesis that ILP would rescue non-pregnant and late-pregnant rats from bupivicane-induced cardiotoxicity.

## Materials and methods

Protocols received institutional review and committee approval. The investigation conformed to the National Institutes of Health Guide for the Care and Use of Laboratory Animals (NIH Pub. No. 85-23, Revised 1996). Animals were randomly assigned to different experimental groups. Experimenters could not be blinded to experimental conditions due to a clear difference in the appearance between non-pregnant and late-pregnant rats.

### Bupivacaine-induced cardiotoxicity model in rats

#### Animals and treatments

**Figure 1:**
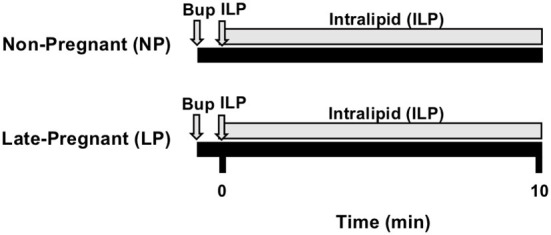
Experimental protocol. Non-pregnant (NP, *n* = 6) and late-pregnant (LP, day 20, *n* = 7) rats were used. Cardiac arrest was induced with bupivacaine (Bup) and immediately followed by resuscitation with intralipid (ILP) bolus followed by infusion and chest compressions for 10-min. EKG was recorded throughout experiment and heart rate (HR), left ventricular ejection fraction (LVEF), and left ventricular fractional shortening (LVFS) were acquired at baseline and 10-min.

We used the well-established bupivacaine-induced cardiotoxicity model in rats as previously described in our publications (2, 3). Briefly, adult female (200–300g) non-pregnant (NP, diestrus, *n* = 6) and late-pregnant (LP, day-20 of pregnancy, *n* = 7) Sprague Dawley rats were anesthetized with isoflurane (2–3%). Rats were placed in an anesthetic chamber with 2–3% isoflurane and once unconscious, they were placed on an ultrasound platform in the supine position breathing 1.5% isoflurane *via* a tube covering their nose and mouth until tracheostomy was performed. Tracheostomy was performed using a 16-gauge angio-catheter and rats were ventilated with a rodent ventilator (Harvard Apparatus) in supine position with a tidal volume of 4 ml/kg and an inspiratory:expiratory (I:E) ratio of 1:2. Femoral vein was accessed through a 24-gauge intravenous catheter. Body temperature was maintained at 37°C. Rats received a single bupivacaine bolus (10 mg/kg, IV over ~20 seconds) to induce asystole. Resuscitation with 20% ILP (5-ml/kg actual body weight single bolus, and 0.5-ml/kg/min maintenance) and chest compressions were continued for 10 min by the same investigator to minimize variability within and between groups. Serial B-Mode and M-Mode transthoracic echocardiography was continuously performed using a Vevo2100 system (VisualSonics) equipped with a 30-MHz linear transducer. Standard Lead II electrocardiograms (EKG) were acquired under anesthesia continuously throughout the experiment. The heart rate (HR, beats per min, bpm), left ventricular ejection-fraction (LVEF%), and left ventricular fractional shortening (LVFS%) were calculated at baseline and 10 min after ILP treatment (Figure 1). Baseline values are defined as the values prior to the administration of bupivacaine.

### Statistical analysis

A sample size of non-pregnant (*n* = 6) vs. late-pregnant (*n* = 7) rats, allows us to adequately detect standardized effect size as small as 2.0 (80% power, *t-*test, two-tailed, alpha = 0.05). HR, LVEF, and LVFS were compared within each experimental group from baseline to 10-min using a paired samples *t*-test. Data are expressed as mean±SD followed by 95% CI. *P*-values < 0.05 were considered statistically significant.

## Results

### Intralipid successfully rescues bupivacaine-induced cardiac arrest in non-pregnant rats

**Figure 2:**
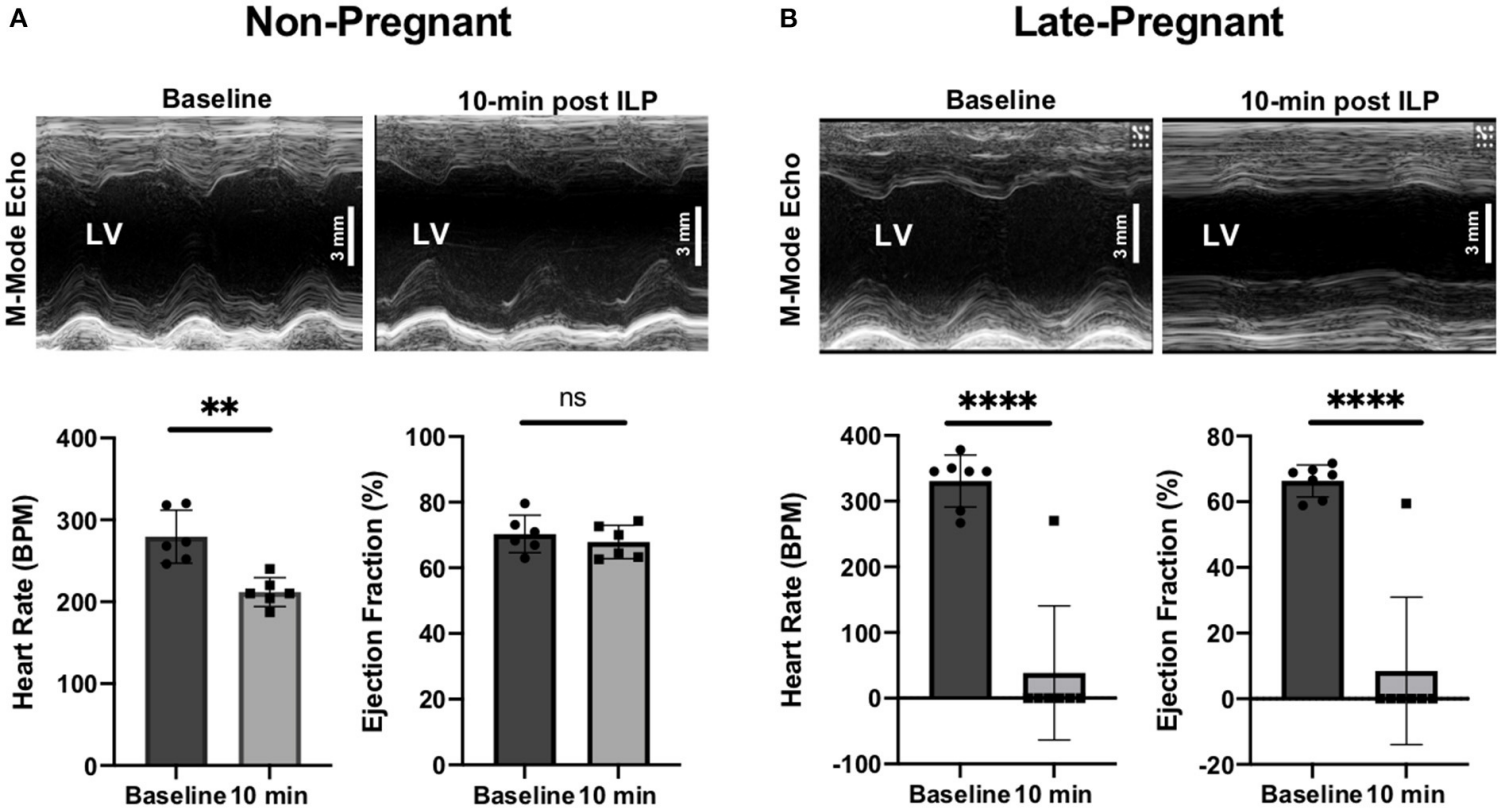
M-Mode transthoracic echocardiographs at baseline and 10-min after intralipid (ILP) bolus showing left ventricular ejection fractions (LVEF, %) and heart rate (beats per minute, bpm) at baseline and 10-min after ILP bolus in non-pregnant (NP) **(A)** and late-pregnant (LP) **(B)** rats. **P-value < 0.01; ****P-value < 0.0001; ns = non-significant.

All non-pregnant rats were rescued by ILP. Rats had a HR of 280 ± 32 bpm (95% CI 254, 305) at baseline vs. 212 ± 18 bpm (95% CI 198, 226) at 10-min post ILP (*p* < 0.01). LVEF was 70 ± 6% (95% CI 66, 75) at baseline vs. 68 ± 5% (95% 64, 72) at 10-min post ILP (*p* = ns), and LVFS was 41 ± 5% (95% CI 37, 45) at baseline vs. 39 ± 4% (95% CI 35, 42) at 10-min post ILP (*p* = ns) (Figure 2A).

### Intralipid fails to rescue bupivacaine-induced cardiac arrest in late-pregnant rats

All late-pregnant rats developed cardiac arrest within a few seconds after a toxic dose of bupivacaine. Contrary to the hypothesis, 6 out of the 7 rats were not rescued by ILP with the appropriate rescue dose (ml/kg actual body weight). In the late-pregnant rats (*n* = 7) the average baseline HR, LVEF and LVFS were 330 ± 40 bpm (95% CI 301, 360), 66 ± 5% (95% CI 63, 70) and 38 ± 4% (95% CI 35, 40), respectively (Figure 2B). At 10-min after ILP, the HR, LVEF, and LVFS were 39 ± 102 bpm (95% CI −37, 114) (*p* < 0.0001 vs. baseline), 8 ± 22% (95% CI −8, 25) (*p* < 0.0001 vs. baseline), and 5 ± 12% (95% CI −4, 14) (*p* < 0.001 vs. baseline), respectively.

## Discussion

Our previous work has demonstrated that ILP protects the heart against ischemia/reperfusion (I/R) injury and bupivacaine-induced cardiotoxicity (2, 3, 6–8). In the current study, we tested the hypothesis that an appropriate dose of ILP would rescue late-pregnant rats from bupivacaine-induced cardiotoxicity. We demonstrate for the first time that ILP successfully rescues bupivacaine-induced cardiac arrest in non-pregnant female rats, however, it fails to rescue bupivacaine-induced cardiac arrest in late-pregnant rats.

This failure of ILP rescue of bupivacaine-induced cardiac arrest in late-pregnant rats is most likely multi-factorial. Physiologic changes associated with pregnancy, including mechanical, hormonal, and biochemical changes, alterations in sensitivity to bupivacaine, pregnancy-induced heart hypertrophy and changes in cardioprotective signaling cascades (10) may be responsible for the failure of ILP to reliably rescue bupivacaine-induced cardiac arrest in pregnancy.

Bupivacaine is one of the most commonly used local anesthetics to provide labor analgesia (11, 12). Clinically, some reports suggest that dose requirements of local anesthetics for epidural anesthesia are reduced in pregnancy (13). Pregnancy may also lead to increased sensitivity to their anesthetic effect, potentiating local anesthetic systemic toxicity (LAST) (13). Pregnancy in laboratory animals and progesterone pre-treatment of cardiac tissue, have been shown to increase the susceptibility of bupivacaine-induced cardiotoxicity (1). One suggested mechanism states that enhanced spread of epidurally administered local anesthetic may be caused by increased pressure in the epidural space as a result of mechanical uterine contractions or by the distention of epidural veins (14, 15). However, a subsequent more recent study by Santos et al. did not demonstrate pregnancy-induced potentiation of cardiotoxic effects of bupivacaine and ropivacaine in pregnant ewes (16). Additionally, in contrast to many studies supporting the increased susceptibility of bupivacaine in pregnant female rats, Diez et al., found that pregnancy was not associated with an increase in susceptibility to bupivacaine when compared to non-pregnant controls, but appeared to slightly decrease axonal sensitivity (15). They suggested that a likely explanation involves a multitude of factors including alterations in diffusion barriers and activation of endogenous analgesic systems. Therefore, the complexity of physiological changes that occurs during pregnancy may be able to account for the variability in sensitivity to bupivacaine and possibly the lack of ILP-rescue.

The use of lipid emulsion for parturients exhibiting neurologic symptoms of LAST has been described in a few case reports (17, 18), however it has not been extensively studied during pregnancy. In each case, neurologic symptoms resolved with a lipid emulsion bolus with no adverse effects to the neonate. However, in each case, parturients were treated with lipid emulsion before cardiovascular collapse. In addition, the American Society of Regional Anesthesia and Pain Medicine released an updated checklist for the practice advisory of managing LAST in 2017. In this most recent update, lipid emulsion dosing was modified. Instead of weight based dosing, patients over 70 kilograms would receive a bolus of 100 mL of lipid emulsion followed by a lipid infusion of 150–200 mL over 15 to 20 mins (19).

The physiologic changes of pregnancy may explain why the standard dose of ILP to recuse non-pregnant rats failed to rescue the late-pregnant rats. Furthermore, cardiac hypertrophy that occurs in pregnancy may cause resistance to standard lipid emulsion dosing (10). This was demonstrated in a study by Ma et al. that showed cardiac hypertrophy inhibited GSK-3? and the cardioprotective effects of ILP were lost (9). In addition, Moller et al. showed enhanced depressive effects from bupivacaine in isolated rabbit cardiac fibers that were pre-treated with progesterone (1). With declining α-1-glycoprotein levels found during pregnancy, the alteration in protein binding is just another proposed mechanism of these effects.

Our study is not without limitations. The aim of this current brief research report was to determine if ILP would rescue cardiotoxicity in non-pregnant and late-pregnant rats, however, molecular data was not obtained. As previous studies have shown, ILP has been demonstrated to mitigate cardiotoxicity and cardiac injury due to activation of cardioprotective signaling (3, 6, 8, 9). Future studies would need to consider collecting cardiac tissue and observing changes in these cardioprotective mechanisms. For example, pathways specifically targeting the mitochondrial permeability transition pore (mPTP) (3), would be of interest. In addition, the current study questions whether the standard ILP dose will successfully rescue the pregnant patients with bupivacaine-induced cardiac arrest, especially considering the recent changes in lipid emulsion dosing recommendations. Going forward, more experiments must be performed to investigate the optimal ILP rescue dosing regimen for bupivacaine-induced cardiotoxicity in late-pregnant rats, as it may well be higher than their non-pregnant counterparts, as well as measuring the concentation of bupivicane in the blood and myocardium at the 10 min mark after the infuison of ILP.

Furthermore, in our study, both the administration of chest compressions and positioning during resuscitation may have served as potential confounders. Chest compressions for both non-pregnant and late-pregnant rats were performed by the same investigator to minimize variability within and between the two groups. Also, late-pregnant rats were kept in a supine position during chest compressions, as previous studies have shown an association between the left-lateral tilt position and decreased quality of chest compressions in pregnant women (20). Nonetheless, data on the optimal positioning for late-pregnant rats undergoing chest compressions is limited. Finally, future studies investigating the effects of different anesthestics on resuscitation of bupivicaine-induced cardiotoxicity by ILP are indicated.

## Conclusions

Our results demonstrate, for the first time, that ILP successfully rescues bupivacaine-induced cardiac arrest in non-pregnant female rats. However, it fails to rescue bupivacaine-induced cardiac arrest in late-pregnant rats with standard ml/kg actual body weight dose. These results may have direct implications on the rescue of pregnant patients with local anesthetic cardiotoxicity. Future studies focused on investigating the mechanistic details and optimal dosing of ILP for a successful rescue of bupivacaine cardiotoxicity in late-pregnant rats are certainly warranted.

## Data availability statement

The raw data supporting the conclusions of this article will be made available by the authors, without undue reservation.

## Ethics statement

The animal study was reviewed and approved by UCLA ARC.

## Author contributions

CS contributed to conception and design of the study, performed experiments, analyzed data, and wrote part of manuscript. MZ analyzed data, performed statistical analyses, and wrote part of manuscript. CC and JH analyzed data and wrote part of manuscript. RH analyzed data and provided intellectual input. ME reviewed the manuscript and provided supervision. NK analyzed data, made a figure, and wrote part of manuscript. SU contributed to conception and design of the study, provided funding and supervision, wrote the manuscript, and critically analyzed the data. All authors contributed to manuscript revision, read, and approved the submitted version.

## Funding

SU is supported by a K08 grant (1K08 HL141995 01) from the National Institutes of Health.

## Conflict of interest

The authors declare that the research was conducted in the absence of any commercial or financial relationships that could be construed as a potential conflict of interest.

## Publisher's note

All claims expressed in this article are solely those of the authors and do not necessarily represent those of their affiliated organizations, or those of the publisher, the editors and the reviewers. Any product that may be evaluated in this article, or claim that may be made by its manufacturer, is not guaranteed or endorsed by the publisher.
